# Relevance of Cone-beam computed tomography on diagnosis and surgical planning of the cementoblastoma

**DOI:** 10.4317/jced.58869

**Published:** 2021-12-01

**Authors:** Kaique-Alberto Preto, David-Bologna Neto, Kellen-Cristine Tjioe, Denise-Tostes Oliveira

**Affiliations:** 1 Department of Surgery, Stomatology, Pathology and Radiology (Area of Pathology), Bauru School of Dentistry, University of São Paulo, Bauru, São Paulo, Brazil; 2 Private Practice, Ourinhos, São Paulo, Brazil

## Abstract

The cementoblastoma is a rare odontogenic tumor occurring in the mandibular molar and premolar of the patients in the second and third decades of life. Despite its typical benign behavior, this tumor may promote local destruction by perforating the cortical bone and displacing the mandibular canal. This case report shows a 31-year-old man with an aggressive cementoblastoma attached to the apex of the mandibular first molar. Cone-beam computed tomography revealed a hyperdense mass connected to the root of mandibular molar surrounded by a hypodense area. Multiplanar reconstructions showed rupture of buccal bone plate and tumor invasion of the mandibular canal roof. The surgical planning included enucleation of tumor with the first and second molars extractions and the diagnosis of cementoblastoma was confirmed by histopathology. This case report emphasizes the contribution of cone-beam computed tomography on diagnosis and appropriate surgical planning of the cementoblastoma.

** Key words:**Cone-Beam computed tomography, odontogenic tumors, diagnosis.

## Introduction

The cementoblastoma remains as a rare odontogenic tumor with less than 300 cases reported in the literature (1). This tumor, originated of the neoplastic cementoblasts, occurs mainly in the mandibular first molar and premolar teeth of the young patients in the second/third decade of life, with similar sex distribution (1,2). Its radiographic features are considered almost pathognomonic, showing a rounded radiopaque mass adhered to the root of vital tooth and circumscribed by a radiolucent halo (2-4).

Despite of typical benign behavior, the cementoblastoma can also behave aggressively. The tumor may promote local destruction by expanding and perforating the adjacent cortical bone, leading to resorption of the surrounding teeth, and, less commonly, displacing the mandibular canal (1-4).

A detailed three-dimensional analysis by cone-beam computed tomography (CBCT) of cementoblastoma might provide the determination of the tumor size, density, presence of root resorption, cortical bone expansion and perforation, in addition to the relationship of the tumor mass with the adjacent anatomic structures (5,6). According to the review of 258 cementoblastomas analyzed by Chrcanovic & Gomez (2017), tumors presenting bone expansion and cortical bone perforation had higher recurrence rate. Therefore, the tomographic analysis of cementoblastoma is critical to identify features that are indicative of higher risk of relapse of the lesion. In addition, the imaging analysis of the tumor is required for surgical planning (7). Although, most of cementoblastomas are treated with conservative surgery including the extraction of involved tooth, an incomplete removal of the tumor can lead to recurrence (2,3,8,9).

This case report shows a 31-year-old man with an aggressive cementoblastoma attached to the apex of the mandibular first molar and causing its resorption and perforation of the buccal bone plate. The importance of cone-beam computed tomography on diagnosis and appropriate surgical planning of the cementoblastoma is discussed.

## Case Report

**Figure 1:**
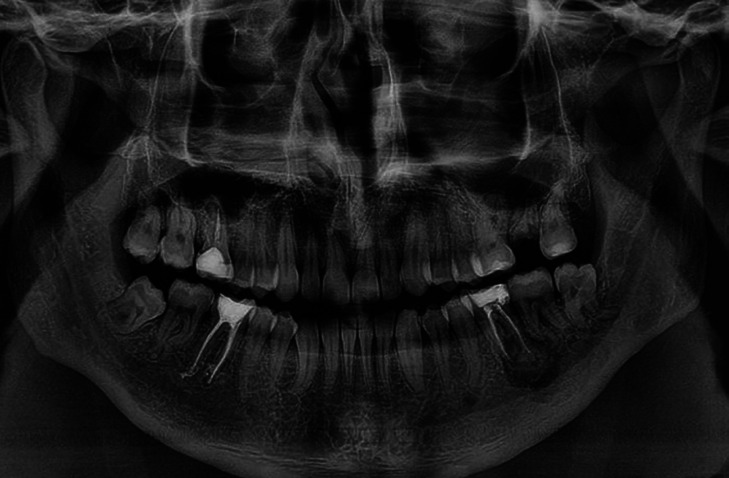
A panoramic radiograph shows the endodontically treated left mandibular first molar presenting a rounded radiopaque mass surrounded by radiolucency area attached to root.

**Figure 2:**
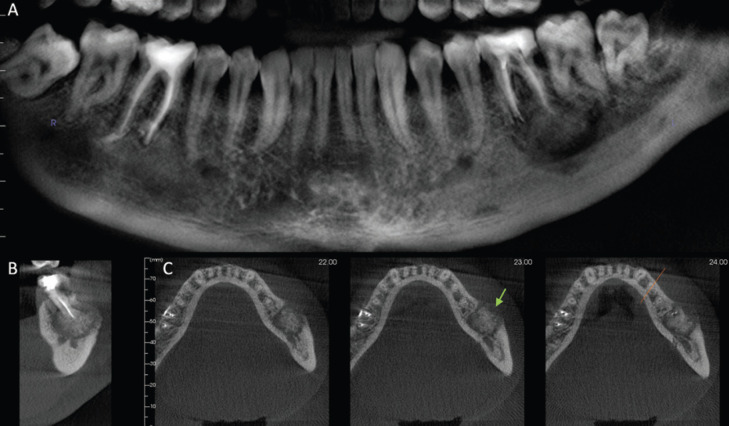
A. Panoramic, B. Cross-sectional, and C. Axial images of cementoblastoma. A. The mixed lesion is attached to the left mandibular first molar and extends to the second molar. B. External resorption of the distal root of the first molar in addition to C. expansion and rupture of the buccal cortical bone plate (green arrow) are observed.

**Figure 3:**
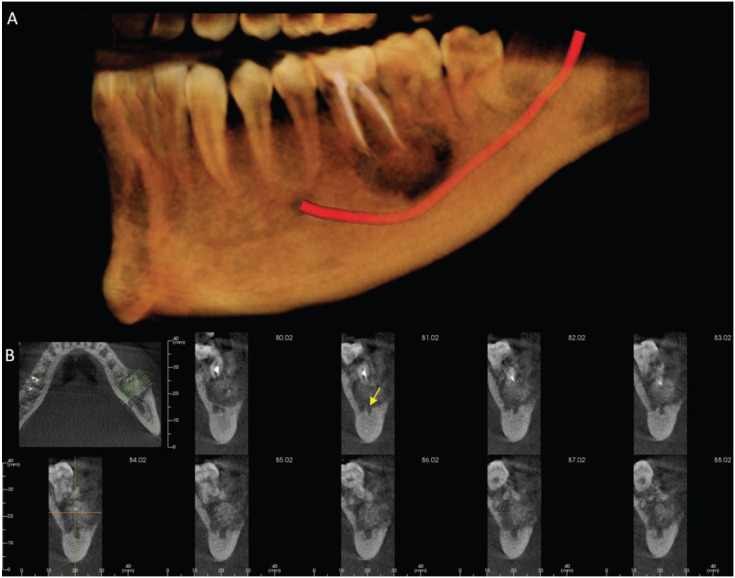
A. 3D imaging evidences the intimate relationship of cementoblastoma with the root of the lower left first molar and with the left mandibular canal (highlighted in red). B. Cross-sectional images exhibiting invasion of the mandibular canal by the tumor (yellow arrow).

**Figure 4:**
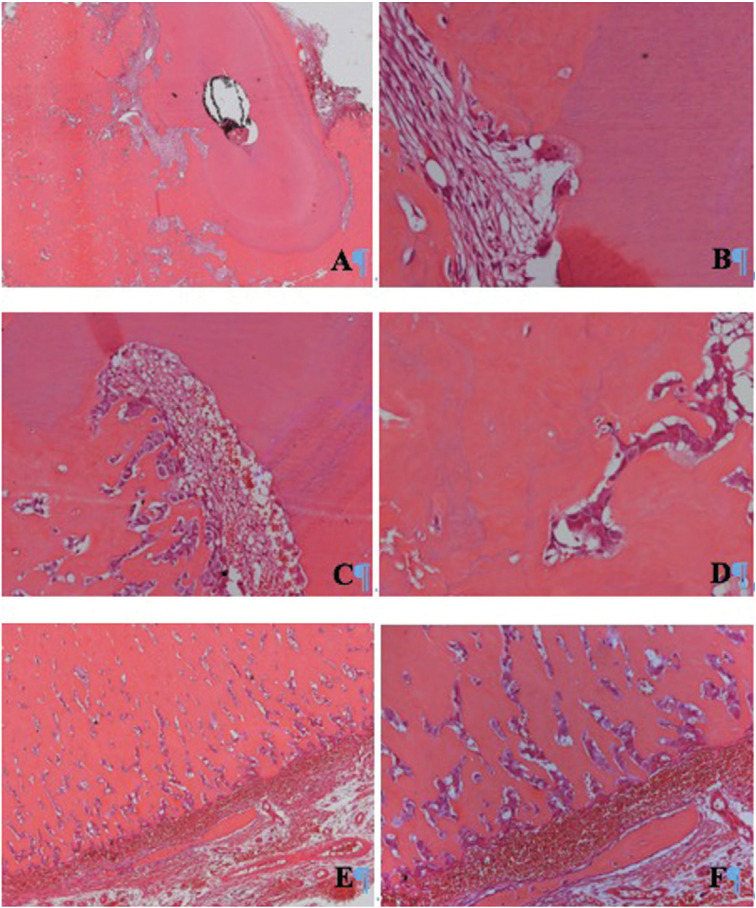
A: Microscopic features of the basophilic tissue cementum-like mass attached to root of the mandibular first molar obliterating the periodontal ligament. B: details of the external radicular resorption containing clusters of multinucleated osteoclast-like cells. C and D: The presence of cementoblast-like neoplastic cells of different sizes within the thick trabeculae with reversal lines in the tumor tissue. E and F: In the periphery of the tumor, radiating columns of uncalcified matrix containing plump active-looking cementoblast supported by fibrovascular stroma were detected. (H&E stain, A/E: original magnification x50, C/F: original magnification x200, B/F: original magnification x400).

A 31-year-old man was referred to the Oral and Maxillofacial surgeon complaining about a persistent and spontaneous pain in the left mandibular first molar (tooth 36). Intraoral examination revealed a hard swelling in the buccal aspect of the referred tooth. The radiographic analysis showed endodontic treatment of the left mandibular first molar. A radiopaque mass was also attached to the apex of the tooth surrounded by radiolucent halo and extending to the mandibular second molar (Fig. 1). A detailed analysis of CBCT showed that the lesion involving the tooth 36 was mixed rather than entirely hyperdense, with irregular borders and fused to the roots (Fig. 2A). Multiplanar CBCT reconstructions revealed obliteration of the periodontal space, external resorption of the distal root of the first mandibular molar, expansion and rupture of buccal cortical bone plate (Fig. 2B,C). The mandibular canal was traced in three dimensional model for analysis of its relationship with the tumor mass (Fig. 3A). Cross sectional images evidenced the invasion of the mandibular canal roof by the tumor (Fig. 3B). The clinical diagnosis was cementoblastoma or osteoblastoma. Surgical excision of the tumor with extraction of left mandibular molars was performed and sent to histopathological analysis. The hematoxylin-eosin stained section revealed an endodontically treated molar tooth with areas of active external radicular resorption presenting clusters of multinucleated osteoclast-like cells (Fig. 4A,B). In addition, a tumor mass fused to root exhibiting basophilic cementoblast-like cells of different sizes within the thick trabeculae with basophilic reversal lines was observed (Fig. 4C,D). In the periphery of the lesion, radiating columns of uncalcified matrix containing hyperchromatic and plump active-looking cementoblast supported by fibrovascular stroma were detected (Fig. 4E,F). The final diagnosis was cementoblastoma. After one year of follow-up, the patient remains asymptomatic and without signs of recurrence.

## Discussion

The cementoblastoma is a rare benign odontogenic tumor with clinical/radiographic and microscopic well established characteristics. This tumor has a high prevalence in the mandibular first molar root of the young patients in the second/third decade of life (1,3,4). All these clinical features were found in our patient and confirmed, recently, in the review of 258 cementoblastomas analyzed by Chrcanovic & Gomez (2017).

Until the 1990s, prior to advent of computed tomography scan, most of the cementoblastomas were diagnosed based on radiographic findings, i.e. rounded radiopaque or mixed-density mass attached to the root of vital tooth causing radicular resorption and obliterating the periodontal ligament space (3,6,12). In the present case, the initial radiography was suggestive of a periapical inflammatory lesion, probably, caused by irreversible pulpitis. Thus, the endodontic treatment of first mandibular molar was performed.

The clinical diagnosis of cementoblastoma based in radiographic findings can be challenging. In early stage, the tumor can be confused with other benign lesions of inflammatory origin that occur in the dental apex such as focal sclerosing osteitis and osteomyelitis (8). Considering the more advanced maturation stages of the cementoblastoma, its radiographic appearance may be indistinguishable from hypercementosis, osteoblastoma and cemento-ossifying fibroma (6,8,10,11). Then, the accurate diagnosis between the aforementioned lesions and the cementoblastoma requires association between histopathology with clinical and imaging exams such as radiography and CBCT (6). Particularly, in the present case, the CBCT enhanced the access to a detailed view of extension, density, and relationship of the cementoblastoma with the adjacent mandibular anatomic structures (Fig. 3).

Due to the continuous tumor growth associated with pain and expansion of bone cortical, our patient was referred to an Oral and Maxillofacial surgeon and a CBCT of the mandibular lesion was requested. The CBCT images obtained in different planes (axial, sagittal, coronal) and 3D reconstruction played a critical role on diagnosis of the cementoblastoma, in its extension in relation to adjacent mandibular second molar, root resorption, and in the detection of bone cortical rupture (Fig. 2). As the cementoblastoma associated with bone plate perforation has a higher risk of relapse of the tumor (1,4,5), its identification on CBCT is crucial. The mandibular canal roof was invaded by the tumor in CBCT analysis (Fig. 3B), and this may be the source of the persistent pain referred by our patient and also described by other authors (6,8).

Additionally, as observed in other cases reported of cementoblastoma (6,7), the three-dimensional CBCT imaging (Fig. 2) provided substantial informations that contributed to appropriate surgical planning of the our patient, including complete removal of the tumor and the decision of the mandibular first and second molars extractions.

The histopathologic analysis of the present case showed distinctive and classical characteristics of the cementoblastoma, according to World Health Organization and described by others (1,2,3,8,10,12). As illustrated in Figure 4, foci of radicular resorption containing clusters of multinucleated osteoclast-like cells and diverse clones of plump active-looking cementoblasts dispersed within the tumor mass containing basophilic reversal lines were observed. These microscopic features reinforce that the survival and proliferative capacity of the neoplastic cementoblasts are not influenced by endodontic treatment, performed previously to enucleation of the lesion. The histopathological characteristics of cementoblastoma may also be shown by osteoblastoma (3,6,8,10,12), however, the identification of tumor attached to the tooth is typical of cementoblastoma and its identification, using radiography or CBCT as performed in present patient (Figs. 1-3), is essential for distinguish both lesions (3,6,11).

Concluding, this case report emphasizes that CBCT contributed for accurate diagnosis of the cementoblastoma enhancing the detailed view of the size, extension, density, the relationship of tumor with adjacent mandibular structures as well as for adequate surgical planning reducing the risk of tumor relapse.
